# Ecological civilization: China's effort to build a shared future for all life on Earth

**DOI:** 10.1093/nsr/nwaa279

**Published:** 2020-11-18

**Authors:** Fuwen Wei, Shuhong Cui, Ning Liu, Jiang Chang, Xiaoge Ping, Tianxiao Ma, Jing Xu, Ronald R Swaisgood, Harvey Locke

**Affiliations:** CAS Key Laboratory of Animal Ecology and Conservation Biology, Institute of Zoology, Chinese Academy of Sciences, China; Center for Evolution and Conservation Biology, Southern Marine Science and Engineering Guangdong Laboratory (Guangzhou), China; Center for Excellence in Animal Evolution and Genetics, Chinese Academy of Sciences, China; Ministry of Ecology and Environment of the People's Republic of China, China; Ministry of Ecology and Environment of the People's Republic of China, China; Chinese Research Academy of Environmental Sciences, China; Endangered Species Scientific Commission of the People's Republic of China, China; CAS Key Laboratory of Animal Ecology and Conservation Biology, Institute of Zoology, Chinese Academy of Sciences, China; Chinese Research Academy of Environmental Sciences, China; San Diego Zoo Global, USA; Beyond the Aichi Targets Task Force, IUCN World Commission on Protected Areas; Yellowstone to Yukon Conservation Initiative, Canada

After hundreds of years of technological revolution and rapid development, humanity has reached a moment of crisis in its relationship with nature [1]. To find better solutions to address global challenges, holistic thinking is required to integrate environmental goals with human activities. Some of these approaches include sustainable development (SD), the planetary boundaries (PB) and, in China, ecological civilization (EC) based on the unity of nature and man (UNM) [2].

The upcoming Convention on Biological Diversity (CBD) COP15 with the theme of  ‘Ecological civilization: building a shared future for all life on Earth’, will review and adopt the post-2020 Global Biodiversity Framework (GBF), and set global goals for biodiversity conservation and sustainable use for the next decade and beyond. We present here the philosophical underpinnings, institutional frameworks and accomplishments of EC in China and its potential contribution to building a shared future for all life on Earth.

## ECOLOGICAL CIVILIZATION AND SUSTAINABLE DEVELOPMENT

**Figure 1.**
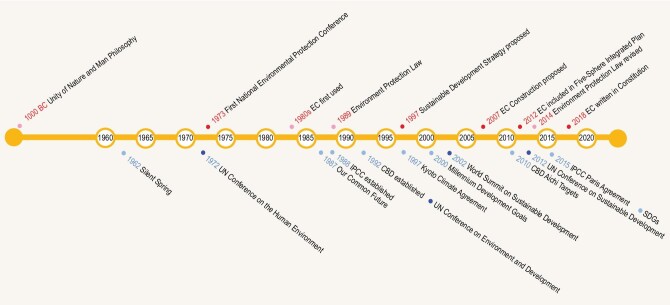
The timeline of the development of the EC concept in China (above) and of SD globally (below).

The term EC was put forth by a European researcher who encouraged self-sacrifice for the benefit of future generations [3]. EC was first used in China in 1980s as an academic concept and widely used in scientific publications since the 2000s. The idea had a strong appeal in China as it was consistent with ancient Chinese Taoist philosophy. As China's economy grew rapidly, it increasingly focused on addressing environmental challenges, and EC was proposed as an innovative way to reconcile economic development and environmental protection at the 17th National Congress of the Communist Party of China in 2007. Since 2012, President Xi Jinping has consistently championed its adoption and maturation, describing it as ‘vital for sustaining the development of the Chinese Nation’. EC was embedded in the country's constitution in 2018, and became the general national development strategy and cornerstone of the ‘New Era’ [4] (Fig. 1).

The Sustainable Development Goals (SDGs) are described by the UN as the blueprint to achieving a better and more sustainable future for all, and address the global challenges related to poverty, inequality, climate change, environmental degradation, peace and justice. While EC and SD both seek to improve humanity's relationship with the environment and have many compatibilities, they are not identical concepts. SD seeks to reconcile the competing interests of society, the economy and the environment to identify the ‘sweet spot’ where they all come together synergistically.

EC has six core principles that are highly compatible with the SDGs. A rich cultural reference, rooted in ancient Chinese knowledge, is used to describe the core EC concept as ‘人与自然和谐共生’ (harmony between man and nature); and the core development principle as ‘绿水青山就是金山银山’ (lucid waters and lush mountains are invaluable assets) [5]. EC and the top-level design and strategic arrangements of the country's ‘Five Sphere Integrated Plan’ can be viewed as the Chinese means of achieving the SDGs. However, unlike the SDGs, there is an additional emphasis on political and cultural aspects, as well as on defining a new relationship between humanity and nature [6].

**Figure 2.**
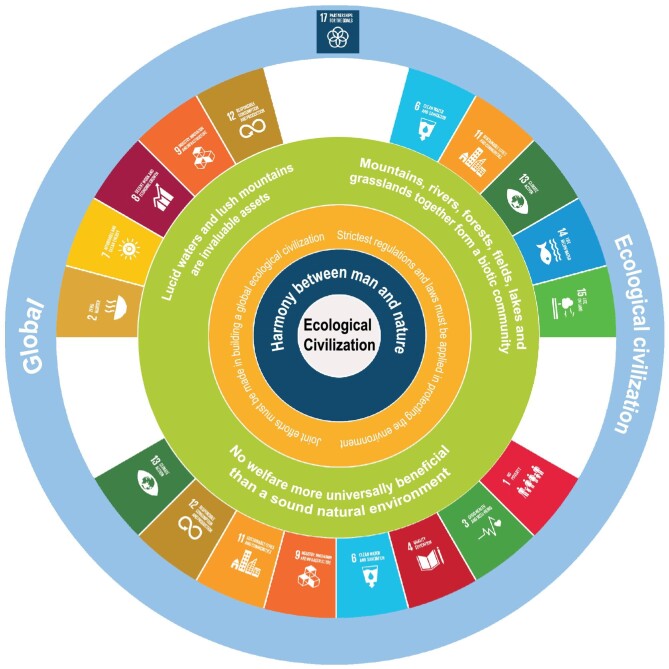
The relationship between six principles of EC and UN Sustainable Development Goals (SDGs). The core concept is ‘Harmony between Man and Nature’, while the strictest regulations and laws, and joint efforts from international communities are safeguarding EC. ‘Lucid waters and lush mountains are invaluable assets’ is the development principle; ‘no welfare more universally beneficial than a sound natural environment’ is the awareness and action principle, while ‘mountains, rivers, forests, fields, lakes and grasslands form a biotic community’ is the governing principle.

International collaboration is seen as essential to achieving the goals of both the SDGs and EC and there is great compatibility between them. Many SDGs are reflected in the policies that have been developed under EC. SDGs 2, 7, 8, 9 and 12 are reflected in policies that aim for innovative, coordinated, green, open and shared development within the carrying capacity of environment, and that consider the needs of future generations by ensuring the sustainability of ecological service provision and maintaining environmental quality. Policies aimed at developing high quality ecological goods to meet people's growing demands for a beautiful environment with wide public participation are congruent with SDGs 1, 3, 4, 6, 9, 11, 12 and 13. The principle that the ecosystem is an integrated entity that needs a systematic project for environmental governance aligns with SDGs 6, 11, 13, 14 and 15. SDGs 5, 10 and 16 will be achieved through the Five Sphere Integrated Plan. All these efforts contribute to global EC which is in line with SDG 17 (Fig. 2).

## EXPERIENCES AND ACHIEVEMENTS OF EC DEVELOPMENT IN CHINA

### Global leadership in afforestation

China launched large-scale national ecological restoration programs, such as the Three-North Shelter Forest Program in 1978, and then two of the largest conservation programs in the world: the Natural Forest Protection Project (1998) banned logging, and the Grain to Green Program (2000) incentivized afforestation. In addition, regional and local ecological restoration and afforestation projects contribute to the ‘greening’ of China. Together, these restoration and afforestation projects have greatly increased forest coverage and ecosystem carbon sequestration, and improved ecosystem services [7]. Recent satellite data (2000–17) shows that China is leading the increase in greening on Earth (25%) with forests contributing to 42% of greening in China [8].

### Promoting biodiversity conservation

While China rapidly industrialized and pursued numerous development projects, it also learned of the need to mitigate developmental impacts on biodiversity [9]. China has rolled out a number of programs to address the biodiversity issues, and the mainstreaming of biodiversity conservation has been embraced throughout all levels of government. In order to strengthen the effectiveness of its Protected Area (PA) system, which covers ∼18% of the country's land, China has initiated reforms to establish a system of protected natural areas with national parks as its mainstay. Other accomplishments include the promulgation of laws, establishment of nature reserve networks, creation of national catalogues of species, assessment of threatened status of vertebrates, higher plants and macrofungi and setting conservation priorities for them; and use of *in situ* and *ex situ* strategies to conserve genetic, species and ecosystem diversity [10]. Many threatened species such as the giant panda, Père David's deer and crested ibis have begun to recover and will likely avoid extinction.

### The Ecological Conservation Redline

A focal point of EC is a national ecological conservation system called the Ecological Conservation Redline (ECR). The ECR was first proposed in 2011, formally adopted in 2017, and the national delimitation on land is expected to be finished by 2020 while efforts to extend it into the marine realm are underway. ECR is designed to constrain human activities in areas important to maintaining national ecological security and which provide essential ecosystem services, including water and soil conservation, biodiversity maintenance, windbreaks and sand-fixation, along with ecologically fragile regions that are prone to soil erosion, desertification and salinization. ECR will expand China's protected area system to cover about 25% of China's land. The completed ECR system is expected to protect more than 95% of China's most valuable ecosystems, 100% of animal and plant habitats under state key conservation, 95% of the best natural landscape resources and 210 important river headwaters, all ecologically fragile areas and important for maintaining ecological function [11].

### Fighting pollution

China's rapid industrialization and urbanization have given rise to pollution problems that affect both human health and biodiversity. Concerted efforts have been made since 2013 to improve air, soil and water quality. The Chinese government has issued the Air Pollution Prevention and Control Action Plan, Water Pollution Prevention and Control Action Plan, and Soil Pollution Prevention and Control Action Plan, in 2013, 2015 and 2016, respectively. Specific targets by 2020 and 2030 and specific measures were adopted for each Action Plan. A target of zero growth in chemical fertilizer and pesticide use by 2020 was set in 2015. Better definition of roles and responsibilities across levels of government have improved management and coordinated implementation. With the implementation of these Action Plans, air, water and soil quality in China have improved. For example, 157 of China's 337 prefecture-level cities have met the air quality standard in 2019, and these 337 cities on average enjoy 82% of days with good air quality [12].

### Implementing green development

China has proposed a comprehensive economic-ecological production framework called ‘Gross Economic-Ecological Product Accounting’ (GEEP) in its evaluation of local governments, which will help spark an industrial transformation and shift the economy to resource-saving and recycling, renewable energy and low-carbon emissions. Another approach recently adopted is to transform ecological advantages into economic advantages. The Kubuqi Desert Eco-Economy embodies this new ecological business model. Market-based mechanisms, with cooperation between government and private enterprises, are employed to achieve sustainable development goals, including restoration of degraded land and improvement in the provision of ecological services and products.

### Climate change

China has committed to aiming to achieve carbon neutrality by 2060 and is setting a pathway to achieve it. China has already become a leader in the research, development, production and application of clean energy technologies. While emissions overall increased, China's carbon emission intensity decreased by about 45.8% from 2005 to 2018, exceeding the target of 40%–45% reduction by 2020, and reducing carbon dioxide emissions by more than 4 billion tons [13]. It is also exploring nature-based solutions to climate change.

## LESSONS LEARNED THAT COULD HELP BUILD A SHARED FUTURE FOR ALL LIFE ON EARTH

As the host of CBD COP15, China has expressed its determination to make commitments and contributions to global biodiversity conservation, and work with other countries to explore a successful paradigm for a harmonious relationship between human beings and nature. The lessons learned in China to date may be helpful in informing national and global efforts.

### Redefine the relationship between humanity and nature

The anthropocentric paradigm developed about two centuries ago, which holds an exploitative attitude toward nature, has significantly contributed to the present ecological crisis. China has recognized that a systematic understanding of the relationship between human beings and nature, and a fundamental shift from viewing humans as isolated in a competitive world, to seeing themselves as an integral part of an interconnected society and biosphere, is needed [2]. At the UN Summit on Biodiversity 2020, President Xi Jinping said: “China has pursued development under the vision of building an ecological civilization. From the traditional Chinese wisdom that the laws of Nature govern all things and that Man must seek harmony with Nature, to the new development philosophy emphasizing innovative, coordinated, green and open development for all … the goal is to seek a kind of modernization that promotes harmonious coexistence of Man and Nature.”

The ancient Taoist idea of the UNM advocates for the intrinsic value of nature, upholding the belief that humanity is a component of nature, and promoting respect for nature and the need to conform to the rule of nature [2]. The EC approach is more than just a pragmatic reliance on ecosystem service values, and instead places inherent value on nature. Other cultures have their own traditions that could be drawn upon to set a national philosophy that seeks the unity of humanity and nature. Such a reconsideration of the relationship between human beings and nature could help the international community to find better solutions regarding environmental challenges and economic development.

### Implement with pilot projects and adaptive governance

Experiences in China prove that pilot projects are a good way to start. During the pilots and trials, priorities, objectives and technologies can be adapted to the situation. Local, provincial and central governments can coordinate thoroughly on development of goals but different priorities and objectives could be set by different local governments to achieve those goals. Such evidence-based and highly adaptive governance has been found to be essential for the success of sustainability programs in China [7].

The planning and implementation process should take into account the actual economic, political, ecological and legal feasibility, as well as the needs of stakeholders at all levels. Timely evaluation and feedback should be incorporated to take necessary measures to balance multiple planning objectives in the natural environment and economic society to reveal and mitigate shortcomings. Furthermore, a comprehensive monitoring system should be established and multi-source data from different fields should be integrated to minimize uncertainties and aid decision-making with the best available empirical evidence. China has also found that both strict supervision and enforcement from the national level are recommended to improve governance and to achieve goals.

### Strengthen information exchange and cooperation

Given pronounced differences among nations in natural resources, economic development and culture, it is not possible to craft a single solution that works well to address all challenges. However, China's experience with EC is an example of how a concerted national effort to mainstream environmental policy with a culturally appropriate philosophy and leadership by the top of government can help to realize objectives of environmental improvement under the Rio Conventions and the SDGs.

Building the community of the future with common goals for humankind will require extensive sharing of information on best practices, sustainable innovation, problem-solving techniques and traditional wisdom; in so doing, each community will draw on the strength of the other and all may thrive and prosper in a nature-rich future. Enhanced inter-community exchanges and cooperation, coupled with mutual respect for differences will be important in crafting appropriate solutions that meet shared global goals.

## FUTURE RESEARCH AND CONCLUSION

The COVID-19 pandemic has demonstrated that we are inextricably connected with the ecosystems in which we all live, and that our future hangs on establishing a new and more harmonious relationship with nature. Serving as an agent for transformative change, the concept and actions of EC in China provide important insights for the international community to consider when meeting these unprecedented challenges. Ambitious, science-based, measurable, realistic, unambiguous and scalable plans that allow the translation of targets into actionable policies are urgently needed [14].

There is still much to learn. Further research is needed regarding market-oriented eco-compensation mechanisms, consumer support for the products of EC-oriented enterprises, social engagement strategies that cultivate awareness and participation in EC, and the synergies between different goals or targets like biodiversity conservation, climate change, desertification and SDGs [15].

CBD COP 15 provides a good opportunity for discussion of the potential for developing a global eco-civilization appropriate to the diverse cultures of the world. There might be chances for scholars, practitioners and policy-makers worldwide to understand the Chinese experience with EC and generate broader global applications, especially for those countries with very different governance arrangements. China wishes to promote collaboration among international communities and engage in information exchange and sharing of lessons learned with other countries in an atmosphere of mutual respect. China's experiences can stimulate a global conversation about how best to achieve the SDGs and the goal of living in harmony with nature in a manner appropriate to differing national contexts. Such an approach could contribute to the development of a robust new GBF designed to build a shared future for all life on Earth.
